# Evaluation of Patients with Parkinson's Disease in Intensive Care Units: A Cohort Study

**DOI:** 10.1155/2021/2948323

**Published:** 2021-11-05

**Authors:** Álvaro Réa-Neto, Bruna C. Dal Vesco, Rafaella S. Bernardelli, Aline M. Kametani, Mirella C. Oliveira, Hélio A. G. Teive

**Affiliations:** ^1^Center for Studies and Research in Intensive Care Medicine–CEPETI, Curitiba 82530-200, Brazil; ^2^Internal Medicine Department, Hospital de Clínicas, Federal University of Paraná, Curitiba 80060-900, Brazil; ^3^Movement Disorders Unit, Neurology Service, Internal Medicine Department, Hospital de Clínicas, Federal University of Paraná, Curitiba 80060-900, Brazil; ^4^Neurology Diseases Group, Postgraduate Program of Internal Medicine, Hospital de Clínicas, Federal University of Paraná, Curitiba 80060-900, Brazil

## Abstract

**Background:**

Parkinson's disease affects approximately 1% of the worldwide population older than 60 years. This number is estimated to double by 2030, increasing the global burden of the disease. Patients with Parkinson's disease are hospitalized 1.5 times more frequently and for longer periods than those without the disease, increasing health-related costs.

**Objective:**

To compare the characteristics and outcome of patients with and without Parkinson's disease admitted to intensive care units (ICUs).

**Methods:**

Historical cohort study of ICU admissions in a Brazilian city over 18 years. All patients with Parkinson's disease identified were matched for age, sex, year, and place of hospitalization with patients without the disease randomly selected from the same database.

**Results:**

The study included 231 patients with Parkinson's disease (PD group) and 462 controls without the disease (NPD group). Compared with patients in the NPD group, those in the PD group were more frequently admitted with lower level of consciousness and increased APACHE II severity score but required less frequently vasoactive drugs. In total, 42.4% of the patients in the PD group were admitted to the ICUs due to sepsis or trauma. Although these patients had longer hospital stay, the mortality rates were comparable between groups. Parkinson's disease was not associated with mortality, even when controlled for associated factors of disease severity.

**Conclusion:**

Although patients with Parkinson's disease were admitted with higher severity scores and remained in the ICU for a longer time, their mortality rate was not higher than that in patients without the disease.

## 1. Introduction

Parkinson's disease is one of the most prevalent degenerative disorders of the central nervous system [1]. It affects approximately 1% of the worldwide population older than 60 years and, added to population aging, imposes a high economic and social burden [2]. A meta-analysis of 47 studies evaluating the prevalence of Parkinson's disease has observed an increasing prevalence with advancing age, reaching 1903 patients for every 100 thousand individuals older than 80 years and affecting mostly men [1]. The number of persons with Parkinson's disease is estimated to double by 2030, increasing the global burden of the disease [3].

Parkinson's disease is responsible for a large percentage of loss of disability-adjusted life years (DALYs) [4]. As well as dementia syndromes, Parkinson's disease may be related to the worsening of other diseases and, therefore, increasing morbidity and mortality [5, 6]. Patients with Parkinson's disease are hospitalized approximately 1.5 times more frequently and for 2–14 days longer than those without the disease, increasing costs and use of healthcare resources [7–9].

Considering the aging of the population, the increased life expectancy of patients with Parkinson's disease, and the scarcity of recent multicenter data on patients with the disease admitted to intensive care units (ICUs), we designed the present study to analyze the course of these patients after admission to ICUs. To accomplish that, we compared the characteristics of hospitalization and outcome between patients with and without Parkinson's disease in ICUs, thereby contributing to the improvement of care and healthcare for these patients and to the development of strategies to prevent harm and reduce the morbidity associated with the disease.

## 2. Materials and Methods

Historical cohort study of patients admitted to ICUs across seven hospitals in Curitiba (Paraná, Brazil). The data were extracted from the database of the Center for Study and Research in Intensive Care (CEPETI), which has been updated since 2000 with all data of patients consecutively admitted to the ICUs. This study was performed in accordance with the Declaration of Helsinki. The study project was approved by the Human Research Ethics Committee of the Neurology Institute of Curitiba (INC) (protocol number 3573668).

All records of patients with Parkinson's disease admitted to the ICUs between January 2001 and August 2019 (PD group) were selected from a database using the following descriptors: “Parkinson,” “G20-Parkinson's Disease,” and “F02.3-Dementia in Parkinson's Disease” in the “admission ICD-10,” “comorbidities,” and “discharge summary” fields. We excluded those records that were duplicated, had other diagnoses, or had only the word “Parkinson” in the ICD-10 code such as atypical, secondary, and heredodegenerative Parkinsonism, Wolff–Parkinson–White, and poisoning or accidental intoxication due to exposure to anticonvulsants (antiepileptics), sedatives, hypnotics, antiparkinsonian drugs, or psychotropics.

Records of patients without Parkinson's disease (NPD group) were randomly selected from the same database and paired for age, sex, year, and hospital of admission with those in the PD group in a 2 : 1 ratio. The software R (R Foundation for Statistical Computing, Vienna, Austria) was used to select the patients in the NPD group for pairing with those in the PD group.

We collected the following variables: age, sex, source of transfer to the ICU, public or private healthcare, reason for ICU admission, hemodynamic support with vasoactive drugs and invasive ventilatory support on ICU admission, Sequential Organ Failure Assessment (SOFA) and Acute Physiology and Chronic Health Evaluation (APACHE II) scores in the first 24 hours in the ICU, level of consciousness measured by the Glasgow coma score on ICU admission and discharge, level of advanced life support, length of ICU stay, and ICU mortality.

### 2.1. Statistical Analysis

Dichotomous categorical variables (i.e., sex, public or private health care, hemodynamic support with vasoactive drugs and invasive ventilatory support on ICU admission, and mortality) were described by absolute and percentage frequency and compared between groups using Fisher's exact test. The same statistical test was used to compare the proportion of patients who received some advanced life support limitation among those with and without Parkinson's disease, stratified by the outcome in the ICU (discharge or death).

The source of transfer to the ICU and reasons for ICU admission (the latter categorized into eight groups) were described by absolute and percentage frequency and compared between groups using the chi-square test followed by analysis of adjusted residuals.

The age of the patients had a normal distribution and was described using means and standard deviations and compared between groups using Student's *t*-test for independent samples, while the length of ICU stay and APACHE II and Glasgow scores were described using means, medians, and interquartile ranges and compared between groups using the nonparametric Mann–Whitney test. Glasgow score at admission were dichotomized into ≤14 and 15, indicating, respectively, lower and normal levels of consciousness; the results were described using absolute frequency and percentage and compared between groups using Fisher's exact test.

The analysis of the association between mortality and Parkinson's disease was controlled by each variable that differed significantly between the PD and NPD groups. This association was analyzed after stratification according to each of the eight groups of reasons for ICU admission and for the variable “source of transfer to the ICU” using the chi-square test. Multivariate logistic regression analysis, described as odds ratios (ORs) and 95% confidence intervals (CIs), assessed the influence of Parkinson's disease on ICU mortality controlled for variables that differed significantly between the PD and the NPD groups in the hypothesis tests and that were related to the death outcome in the univariate regression analysis.

The level of statistical significance was set at 5%, and the data were analyzed using the statistical software Stata, version 15.0 (StataCorp LLC, College Station, TX, USA). Imputation for missing data was not performed.

## 3. Results

**Figure 1:**
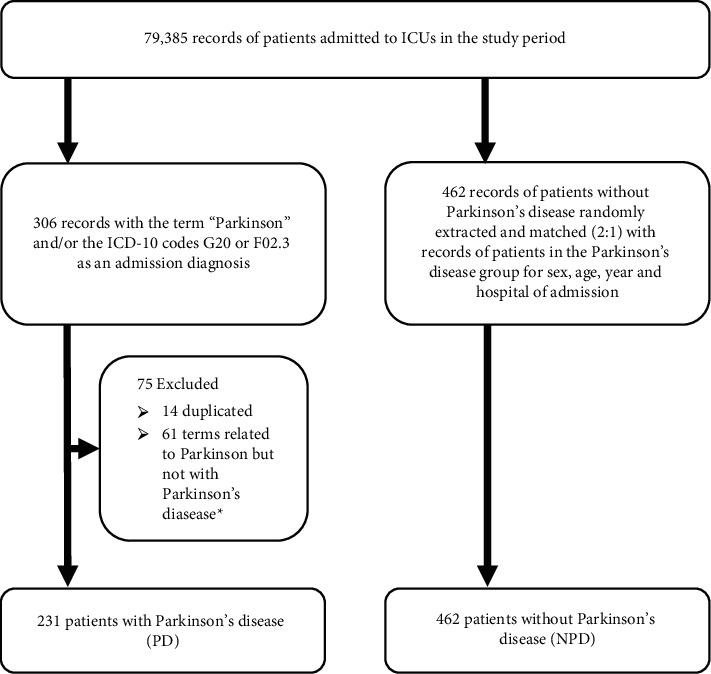
Sample selection flowchart. ^*∗*^Records with the following terms related to Parkinson but not to Parkinson's disease were excluded: “secondary Parkinsonism,” “preexcitation syndrome (Wolff–Parkinson–White syndrome),” “accidental poisoning by and exposure to antiepileptic, sedative-hypnotic, anti-Parkinsonism, and psychotropic drugs, not elsewhere classified,” “intentional self-poisoning by and exposure to antiepileptic, sedative-hypnotic, anti-Parkinsonism, and psychotropic drugs, not elsewhere classified,” and “poisoning by and exposure to antiepileptic, sedative-hypnotic, anti-Parkinsonism, and psychotropic drugs, not elsewhere classified, undetermined intent.”

Of 79,385 ICU admissions identified in the CEPETI database during the study period, 306 were related to the term “Parkinson.” Of these, 75 were excluded due to duplicate records or other diagnoses citing the term “Parkinson” and/or “Parkinsonism,” in which the diagnosis of Parkinson's disease was not mentioned, yielding 231 records of patients with Parkinson's disease (PD group). These records were matched with 462 records of patients without Parkinson's disease (NPD group) (Figure 1).

**Figure 2:**
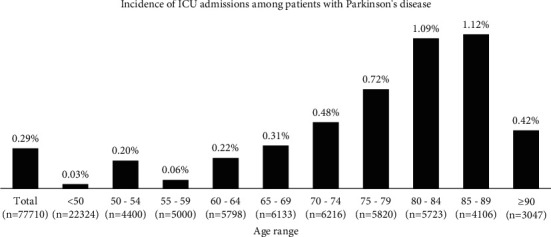
Total incidence of hospitalizations between 2000 and 2019 of patients with Parkinson's disease admitted to the intensive care units (ICUs) included in the study, stratified by the age group. A total of 1304 patients younger than 18 years and 371 with no record of age in the database were not included in the analysis.

The total incidence of hospitalizations of patients with Parkinson's disease in the ICUs was 0.3%. The age group with the highest incidence of the disease was between 80 and 89 years (Figure 2).

The total number of hospitalizations among patients with and without Parkinson's disease was 693. The mean age was 77.7 ± 9.60 years and 50.2% were men. Only 24.2% of the patients received public healthcare. Most patients hospitalized were referred from the emergency department (45.2%). In total, 49.2% of the ICU admissions were due to sepsis or elective surgery, while 56.2% had some organ dysfunction on ICU admission involving a lower level of consciousness or need for vasoactive drugs and/or invasive ventilatory support. The median APACHE II severity score was 16, and the median length of ICU stay was 3 days (range 1–62 days). In all, one-fifth of the patients (DP and SDP groups) died during hospitalization in the ICU.

The PD group had a significantly greater percentage of hospitalizations related to sepsis, trauma, and neurological conditions than the NPD group, in which predominated patients who had undergone elective surgery (*p* < 0.001 for the comparison among all eight reasons for ICU admission). Likewise, the PD group had a higher prevalence of patients coming from emergency services and wards (Table 1).

On ICU admission, patients in the PD group had more frequently a lower level of consciousness (lower Glasgow score) and increased severity (APACHE II score) compared with those in the NPD group, although the NPD group required vasoactive drugs more frequently. There were no significant differences between groups regarding invasive ventilatory support or in SOFA score within the first 24 hours in the ICU (Table 1).

The PD group had a longer ICU stay (*p*=0.027) compared with the NPD group, while the mortality rates between both groups showed no difference. Most patients who died had some degree of limitation of advanced life support, but no difference between the groups was observed in this regard. More patients with Parkinson's disease were discharged from the ICU with some degree of limitation of advanced life support, and the Glasgow score at discharge was lower in these patients (*p* < 0.001) (Table 1).

Variables showing significant differences between the PD and NPD groups were analyzed as prognostic factors for mortality using univariate logistic regression analysis. In this analysis, Parkinson's disease showed no relationship with mortality, while the use of vasoactive drugs and lower level of consciousness on ICU admission, as well as higher APACHE II scores, increased the chances of death (Table 2). On multivariate analysis, Parkinson's disease showed no association with death after control for lower level of consciousness on ICU admission (OR 0.65, 95% CI 0.42–1.00, *p*=0.05), hemodynamic support with vasoactive drugs on ICU admission (OR 1.04, 95% CI 0.68–1.59, *p*=0.867), and APACHE II score in the first 24 hours in the ICU (OR 0.70, 95% CI 0.43–1.12, *p*=0.138) (Table 2).

**Figure 3:**
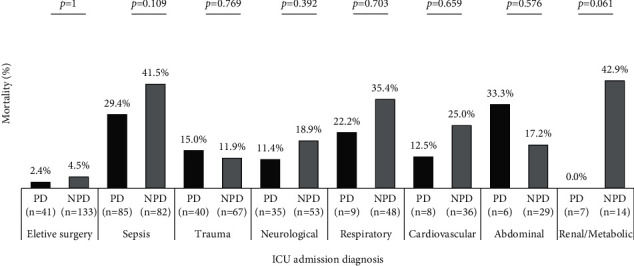
Comparison of mortality rates between patients with and without Parkinson's disease (PD and NPD groups, respectively) according to the reason for admission to the intensive care unit (ICU). The results are presented in percentages of deaths considering the total number of patients admitted according to the reason for ICU admission stratified by the group. The *p* values >0.006 refer the significance adjusted of the chi-square test result. *n*, total number of patients in each group categorized by reason for ICU admission.

**Figure 4:**
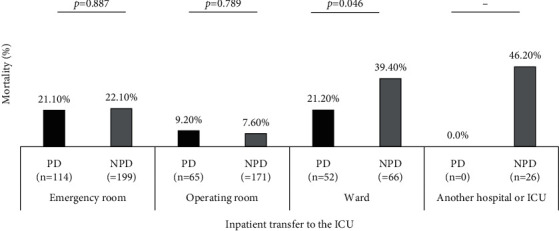
Comparison of mortality rates between patients with and without Parkinson's disease (PD and NPD, respectively) according to the source of transfer to the intensive care unit (ICU). The comparison was not performed in the group “another hospital or ICU,” since no patients in the PD group were transferred from another ICU or hospital. The results are presented in percentage of deaths considering the total number of patients admitted according to the source of transfer to the ICU stratified by the PD/NPD group. The *p* values >0.012 refer the significance adjusted of the chi-square test result.

A comparison of mortality rates according to reasons for ICU admission showed no significant differences between the PD and NPD groups (Figure 3). No significant differences in mortality rates were observed between groups categorized by the source of transfer to the ICU (Figure 4).

## 4. Discussion

Among patients with Parkinson's disease in our study, 16.5% were admitted to the ICUs due to morbidity directly related to the disease, while 54% were admitted due to sepsis or trauma. This finding is in line with literature data showing that at least half of the patients with Parkinson's disease are not admitted to hospitals due to direct complications of the disease, but rather due to indirect causes (pneumonia, urinary tract infection, and falls) that are also responsible for the higher mortality of these patients in ICUs [3, 10–12].

A recent study carried out in India has shown that 34.9% of the patients with Parkinson's disease in a neurology ward were admitted to the ICU and that the ICU admissions were not directly related to the primary disease but to indirect factors related to the disease. Pneumonia was the main cause of hospitalization (in 16% of the patients), followed by urinary tract infection (12%) [12]. Similarly, a prospective Brazilian study in a cohort of 230 patients with Parkinson's disease followed up for 13 months found that 5.6% of the patients required emergency care, and two-thirds of the cases were due to infection or fracture of the femur, with a significant worsening of the severity of the disease after hospitalization [11]. As the disease progresses, symptoms such as immobility, dysphagia, and autonomic manifestations appear, increasing the risk of pulmonary aspiration and urinary dysfunction [3, 10]. In addition, the reduced mobility that the patients present at later stages of the disease favors the occurrence of falls [3, 11].

Patients with neurodegenerative diseases become increasingly frail with age, which leads to a rapid deterioration of the patient's condition with any new clinical complication, resulting in longer hospital stay, greater requirement for invasive ventilatory support, and increased risk of complications and death [10]. Although our study showed no differences in use of invasive mechanical ventilation or mortality between groups, patients with Parkinson's disease remained longer in the ICU.

The lower level of awareness on admission and the greater limitations of advanced life support for future hospitalizations on discharge, found more frequently in patients with Parkinson's disease, are probably associated with the natural progression of the disease, resulting from cognitive decline, mood disorders, and frailty in these patients who often develop delirium or organ dysfunction secondary to the condition that led to admission to the ICU [12]. To date, there are no treatments that can interrupt or delay the course of the disease, leading to important cognitive and functional decline [13]. So, more and more patients, families, and health professionals are presented with the possibility of palliative care as the disease becomes more severe [14]. Besides, some aspects of medical care can be very aggressive and result in futile treatment to patients in advanced condition in their end of life [5]. The right of patients and families to align an advance care planning to define the goals of care for the end of their life can reduce fear and anxiety and bring them more satisfaction [15].

The lack of a significant association between Parkinson's disease and mortality, observed in the present study, has also been reported in a Canadian study, in which the mortality rate of patients with Parkinson's disease remained unchanged over a 6-year follow-up period, suggesting that the risk of death is not related to the duration of the disease [16]. Additionally, an observational study conducted in India found no relationship between the duration and severity of Parkinson's disease and the need for ICU admission [12].

Although our study is one of few to have compared the profile of patients with and without Parkinson's disease admitted to ICUs, it has some limitations. We did not assess how long the patients admitted to the ICU had Parkinson's disease and how severe the disease was. Additionally, the groups were not paired for other comorbidities commonly seen in older individuals. Both these aspects can influence the morbidity and mortality of patients with Parkinson's disease. In addition, although some baseline characteristics were unbalanced between groups at baseline, the relationship of Parkinson's disease to mortality was controlled for these characteristics by multivariate statistical analysis and subgroup analyses.

## 5. Conclusions

In conclusion, our study showed that patients with Parkinson's disease compared with those without the disease are more frequently admitted to ICUs due to sepsis, have more frequently lower level of consciousness and higher APACHE II score, require less frequently vasoactive drugs, and remain in the ICU for a longer time. However, mortality was not higher in these patients compared with those without the disease, even after stratification for reasons of hospitalization or control for variables of disease severity during hospitalization. However, these patients are also discharged with greater limitation in advanced life support and a more frequent lower level of consciousness.

## Figures and Tables

**Table 1:** Epidemiological and clinical characteristics of the study groups.

Variables	PD (*n* = 231)	NPD (*n* = 462)	*P* value
Admission characteristics			
Male gender, *n* (%)	116 (50.2)	232 (50.2)	1^*∗*^
Age, mean ± SD	77.7 ± 9.6	77.9 ± 9.6	0.837^*∗∗*^
Private healthcare, *n* (%)	171 (74.0)	354 (76.6)	0.453^*∗*^
Source of transfer to the ICU, *n* (%)			
Emergency room	114 (49.4)	199 (43.1)	<0.001^*∗∗∗*^
Surgical room	65 (28.1)	171 (37.0)	
Ward	52 (22.5)	66 (14.3)	
Another hospital or ICU	0 (0.0)	26 (5.6)	
Reason for admission, *n* (%)			
Neurological	35 (15.2)	53 (11.5)	<0.001^*∗∗∗*^
Abdominal	6 (2.6)	29 (6.3)	
Cardiological	8 (3.5)	36 (7.8)	
Respiratory	9 (3.9)	48 (10.4)	
Renal/metabolic	7 (3.0)	14 (3.0)	
Trauma	40 (17.3)	67 (14.5)	
Elective surgery	41 (17.7)	133 (28.8)	
Sepsis	85 (36.8)	82 (17.7)	

Clinical condition on ICU admission			
Lower level of consciousness^#^, *n* (%)^a^	163 (73.1)	192 (43.9)	<0.001^*∗*^
Glasgow score, median (IQR)	14 (11–15)	15 (13–15)	<0.001^*∗∗∗∗*^
Requirement of hemodynamic support with VAD, *n* (%)^b^	14 (6.28)	57 (13)	0.008^*∗*^
Requirement of IMV, *n* (%)^a^	44 (19.7)	77 (17.6)	0.507^*∗*^
APACHE II score in the first 24 hours, median (IQR)	17 (12–23)	15 (11–22)	0.007^*∗∗∗*^
SOFA score in the first 24 hours, median (IQR)^c^	4 (2–6)	3 (2–5)	0.118

ICU outcome variables			
Days of hospitalization, median (IQR)	3 (2–8)	3 (1–6)	0.027^*∗∗∗*^
Mortality, *n* (%)	41 (17.7)	95 (20.6)	0.219^*∗*^
Death with some level of ALS limitation, *n* (%)^d^	25 (86.2)	45 (69.2)	0.123^*∗*^
Discharge with some level of ALS limitation, *n* (%)^e^	22 (15.2)	24 (8.6)	0.038^*∗*^
Glasgow score at discharge, median (IQR)	14 (12–15)	15 (14–15)	<0.0018^*∗∗∗*^

PD, group with Parkinson's disease; NPD, group without Parkinson's disease; IMV, invasive mechanical ventilation; VAD, vasoactive drug; ICU, intensive care unit; ALS, advanced life support; SD, standard deviation; IQR, interquartile range. ^#^This variable was dichotomized by Glasgow score at admission, and any score ≤14 indicated lower level of consciousness. Missing data: ^a^8 in the PD group and 25 in the NPD group; ^b^8 in the PD group and 24 in the NPD group; ^c^74 in the PD group and 207 in the NPD group; ^d^16 in the PD group and 28 in the NPD group; ^e^45 in the PD group and 87 in the NPD group. ^*∗*^Significance of Fisher's exact test. ^*∗∗*^Significance of Student's *t*-test. ^*∗∗∗*^Significance of the chi-square test. ^*∗∗∗*^Significance of the Mann–Whitney test.

**Table 2:** Univariate and multivariate analysis using death as a dependent variable.

Univariate analysis	OR (95% CI)^*∗*^	*P* value
Parkinson's disease	0.83 (0.55–1.25)	0.38
Lower level of consciousness on ICU admission	2.69 (1.76–4.09)	<0.001
VAD on ICU admission	5.81 (3.47–9.73)	<0.001
APACHE II in the first 24 hours in the ICU	1.17 (1.14–1.21)	<0.001

Multivariate analysis evaluating the influence of Parkinson's disease on death	OR (95% CI)^*∗∗*^	*P* value
Parkinson's disease controlled by APACHE II score in the first 24 hours	0.70 (0.43–1.12)	0.138
Parkinson's disease controlled by VAD on ICU admission	1.04 (0.68–1.59)	0.867
Parkinson's disease controlled by lower level of consciousness on ICU admission	0.65 (0.42–1.00)	0.050
Parkinson's disease controlled by VAD and lower level of consciousness on ICU admission	0.83 (0.53–1.29)	0.404

PD, Parkinson's disease; VAD, vasoactive drug; ICU, intensive care unit; APACHE II, Acute Physiology and Chronic Health Evaluation. ^*∗*^Odds ratio and 95% confidence interval of univariate logistic regression analysis, with a significance level of 5%. ^*∗∗*^Odds ratio and 95% confidence interval of the multivariate logistic regression analysis, with a significance level of 5%.

## Data Availability

The datasets analyzed during the current study are available from the corresponding author upon request.
